# Associations of sleep behaviors with white matter hyperintensity volume in middle‐aged to older adults

**DOI:** 10.1002/alz.71457

**Published:** 2026-05-05

**Authors:** Madeline Ally, Daniel H. Aslan, M. Katherine Sayre, Pradyumna K. Bharadwaj, Silvio Maltagliati, Matthew D. Grilli, Mark H. C. Lai, Rand R. Wilcox, Yann C. Klimentidis, David A. Raichlen, Gene E. Alexander

**Affiliations:** ^1^ Department of Psychology University of Arizona Tucson Arizona USA; ^2^ Human and Evolutionary Biology Section Department of Biology University of Southern California Los Angeles California USA; ^3^ Evelyn F. McKnight Brain Institute University of Arizona Tucson Arizona USA; ^4^ Department of Neurology University of Arizona Tucson Arizona USA; ^5^ Department of Psychology University of Southern California Los Angeles California USA; ^6^ Department of Epidemiology and Biostatistics Mel and Enid Zuckerman College of Public Health University of Arizona Tucson Arizona USA; ^7^ BIO5 Institute University of Arizona Tucson Arizona USA; ^8^ Department of Anthropology University of Southern California Los Angeles California USA; ^9^ Department of Psychiatry University of Arizona Tucson Arizona USA; ^10^ Neuroscience and Physiological Sciences Graduate Interdisciplinary Programs University of Arizona Tucson Arizona USA; ^11^ Arizona Alzheimer's Consortium Phoenix Arizona USA

**Keywords:** brain aging, dementia risk, sleep quality, UK Biobank, white matter hyperintensity volume

## Abstract

**INTRODUCTION:**

Poor sleep has been associated with elevated dementia risk, potentially related to its cerebrovascular consequences, measured by cerebral white matter hyperintensity (WMH) volume.

**METHODS:**

We examined self‐reported sleep behaviors and prospective magnetic resonance imaging (MRI) WMH volume, measured 8.8 ± 1.7 (mean ± SD) years later, in 23,377 healthy UK Biobank participants. Each sleep behavior was adjusted for demographic, imaging, and clinical covariates (Model 1), as well as vascular health and lifestyle factors (Model 2), and significant sleep behaviors were then mutually adjusted.

**RESULTS:**

In Model 1, all poor sleep behaviors were associated with greater WMH volume. In Model 2, only sleep duration outside 7–9 h (*β *= 0.015, false discovery rate [FDR]*p *= 0.014), increased daytime napping (*β *= 0.018, FDR*p *= 0.008), and greater sleeplessness (*β *= 0.015, FDR*p *= 0.014) were associated with greater WMH volume, with each behavior demonstrating distinct contributions (0.004 ≤ FDR*p*’s ≤ 0.025).

**DISCUSSION:**

Self‐reported sleep behaviors were prospectively associated with greater WMH volume, suggesting a potential sleep‐related pathway influencing vascular brain health and dementia risk.

## Highlights

- Indicators of poor sleep are linked to greater WMH volume among healthy adults
- Sleep duration, napping, and sleeplessness impact brain aging beyond vascular risk
- Sleep may have a unique role in vascular brain health and dementia risk

## 1 BACKGROUND

Given that dementia prevalence is projected to rise substantially in the coming decades,^1^ growing attention has focused on understanding how modifiable lifestyle behaviors may contribute to underlying disease processes. Sleep, in particular, has emerged as an integral factor for healthy brain aging.^2^, ^3^ Evaluating specific behaviors that influence sleep quality, such as duration, difficulties initiating or maintaining sleep, and other sleep disturbances, along with related consequences (i.e., daytime fatigue, napping) offers an opportunity to clarify the role of sleep in brain aging and dementia risk.

Prior research has linked self‐reported poor sleep quality and related behaviors, including increased sleeplessness and both short and prolonged sleep duration, to worse global cognitive functioning in aging cohorts.^3^, ^4^ Prospectively, adults reporting poorer sleep quality are also at increased risk for cognitive decline^5^, ^6^ and Alzheimer's disease and related dementias (ADRD).^5^, ^7^, ^8^, ^9^ Consequences of inadequate sleep, such as more frequent daytime napping and excessive daytime sleepiness, are also associated with poorer cognitive outcomes cross‐sectionally and longitudinally.^4^, ^10^, ^11^, ^12^, ^13^ However, there is evidence to suggest that these relationships may be more complex, as short napping has been associated with reduced risk of cognitive decline,^8^, ^14^, ^15^ highlighting the need to further clarify how specific sleep behaviors influence cognitive and brain health in aging.

Growing evidence has underscored the impact of sleep on cardiovascular health. During sleep, heart rate and blood pressure typically decrease, a process believed to be crucial for long‐term cardiovascular regulation.^16^, ^17^ Poor sleep quality, including short and prolonged sleep duration and sleeplessness, has been associated with greater risk of hypertension, diabetes, and coronary heart disease, as well as cardiovascular and cerebrovascular events, such as myocardial infarction, stroke, and transient ischemic attacks.^16^, ^18^, ^19^, ^20^ Given the well‐established relationship between cardiovascular health and cognitive decline,^21^, ^22^ these findings point to a possible pathway by which poor sleep may influence dementia risk.

One possible link between sleep, cardiovascular health, and cognition may involve cerebral white matter (WM) lesions, measured as white matter hyperintensity (WMH) volume on magnetic resonance imaging (MRI). Although WM lesions are common within the context of healthy aging,^23^ greater WMH burden has been associated with higher risk and greater severity of neurodegenerative disease.^24^, ^25^, ^26^, ^27^ Prior research has highlighted the influence of modifiable lifestyle factors on WM lesion load, with factors reflecting poorer cardiovascular health, such as high blood pressure, elevated body mass index (BMI), and low levels of physical activity, related to greater WMH burden.^28^, ^29^ As poor sleep is similarly linked to cardiovascular health, examining its relationship with WMH volume may clarify how sleep behaviors relate to vascular brain aging.

Although relatively few studies have directly examined sleep quality and WMH volume, initial evidence suggests an association between poorer sleep quality and greater WM lesion load.^30^, ^31^, ^32^ Specifically, lower ratings of global sleep quality, suboptimal sleep duration (i.e., both shorter [≤5–6 h] and longer [≥8–9 h] sleep), reduced sleep efficiency, increased daytime sleep, and snoring have all been associated with increased WMH volume.^30^, ^31^, ^32^, ^33^, ^34^, ^35^, ^36^ Although these findings highlight that both sleep quantity and quality may play a role in vascular brain health, the nature of these associations is not fully understood, and further investigation is needed to evaluate how distinct sleep behaviors relate to WMH accumulation, particularly within the context of healthy aging where early vascular changes may be most detectable.

In the present study, we investigated the association between self‐reported sleep behaviors and prospective WMH volume within the UK Biobank, a large cohort of community‐dwelling middle‐aged to older adults. We first examined these associations by adjusting for relevant demographic, imaging acquisition, and clinical variables. Then, to further evaluate the unique contributions of these behaviors to vascular brain aging, we additionally controlled for common vascular health and lifestyle factors. We hypothesized that behaviors indicative of poorer sleep would be associated with greater WMH volume after accounting for demographic, imaging acquisition, and clinical factors, and that these associations would persist when further adjusting for vascular health and lifestyle factors.

**RESEARCH IN CONTEXT**

1. **Systematic review**: The authors reviewed prior literature on sleep and cognitive and brain health using traditional sources (e.g., PubMed). While previous studies have examined associations between sleep and white matter hyperintensity (WMH) volume, few have comprehensively restricted samples to minimize health‐related confounds, and even fewer have evaluated the distinct contributions of specific sleep behaviors to better understand their differential impacts on brain aging and dementia risk.
2. **Interpretation**: Sleep behaviors indicative of poor sleep were associated with greater WMH volume, even after accounting for common vascular and lifestyle factors. These findings suggest a possible sleep‐related pathway impacting vascular brain health and potentially influencing risk for Alzheimer's disease and related dementias (ADRD).
3. **Future directions**: To clarify causality and directionality of associations between sleep and brain health in aging, future research should incorporate additional objective and subjective sleep measures, examine cardiovascular and metabolic mediators, and employ diverse methodological approaches, including longitudinal, mechanistic, and interventional study designs.

## 2 METHODS

### 2.1 Participants

This study used data from the UK Biobank, a large‐scale biomedical database containing genetic, lifestyle, and health information from community‐dwelling middle‐aged to older adults in the United Kingdom (*n* ≈ 500,000).^37^ The study was approved by the National Health Service and the National Research Ethics Service, and all participants provided written informed consent. The database is composed of an initial data collection (baseline) between 2006 and 2010. A subset of participants (*n* ≈ 100,000) were invited to return for extensive imaging beginning in 2014.^38^ Although follow‐up imaging data collection is still underway, we utilized data from the first imaging visit with collection between 2014 and 2019. The dataset used for the present analyses was downloaded in August 2023. All participants included were required to have complete data on all study and covariate variables.

**FIGURE 1:**
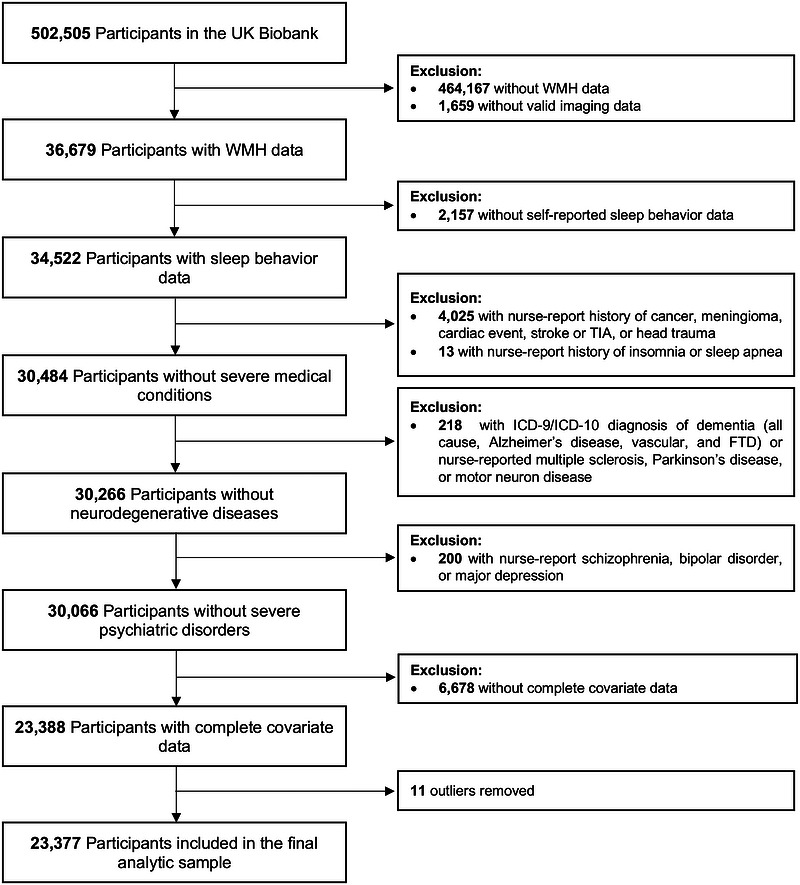
Flow diagram for the final analytic sample. FTD, frontotemporal dementia; ICD‐9, International Classification of Diseases, Ninth Revision; ICD‐10, International Classification of Diseases, Tenth Revision; TIA, transient ischemic attack; WMH, white matter hyperintensity.

To evaluate subclinical variations within a sample of healthy adults, we excluded individuals with major medical, neurological, and psychiatric conditions assessed via available nurse interviews or hospital records up to the time of imaging (Figure 1). Exclusion criteria included a history of cancer, meningioma, cardiac event, stroke or transient ischemic attack, head trauma, or sleep disorders (i.e., sleep apnea and insomnia). Neurodegenerative disease diagnoses (i.e., all‐cause dementia, Alzheimer's disease [AD] dementia, vascular dementia, frontotemporal dementia, multiple sclerosis, Parkinson's disease, or motor neuron disease) were also exclusionary. Furthermore, participants were excluded if they received a psychiatric diagnosis of bipolar disorder, major depression, or schizophrenia.

### 2.2 Sleep behaviors

Self‐reported sleep behaviors were obtained from a touchscreen questionnaire completed at participants’ baseline assessment visit, which have been used previously to evaluate sleep quality in this cohort.^39^ We analyzed responses to five questions to obtain our sleep behaviors of interest: (1) “About how many hours of sleep do you get in every 24 hours? (including naps)” (f.1160) to assess daily sleep duration, (2) “Do you have a nap during the day?” (f.1190) to assess daytime napping, (3) “Do you have trouble falling asleep at night or do you wake up in the middle of the night?” (f.1200) to assess sleeplessness (i.e., subclinical insomnia), (4) “Does your partner or a close relative or friend complain about your snoring” (f.1210) to assess snoring, and (5) “How likely are you to doze off or fall asleep during the daytime when you don't mean to? (e.g., when working, reading, or driving)” (f.1220) to assess daytime dozing. All sleep variables were dichotomized in accordance with previously established procedures^26^, ^39^ and to reduce the influence of any extreme values. Following National Sleep Foundation (NSF) guidelines for adults,^40^ sleep duration was dichotomized into obtaining the recommended 7–9 h of sleep versus sleeping less or more than the recommended range. The remaining sleep variables were collapsed as follows: never/rarely versus sometimes/usually napping, never/rarely versus sometimes/usually having trouble falling asleep, absence versus presence of snoring complaints, and never/rarely versus sometimes/often/all of the time dozing off during the day.

### 2.3 MRI

MRI data using a Siemens Skyra 3T scanner following detailed methods described elsewhere,^38^, ^39^, ^41^ and available online through the UK Biobank's open‐access protocol,^42^ were analyzed. Participants in the present study had available T1‐weighted and T2 fluid‐attenuated inversion recovery (FLAIR) scans acquired between 2014–2019. The available MRI data already removed non‐usable data from initial UK Biobank preprocessing and quality control procedures.^43^ As an additional quality assurance step, we used five QC metrics derived from the structural MRI scans that are provided by the UK Biobank (f.25731‐f.25735) to exclude participants with values greater than 3 standard deviations (SD) from the mean of each variable.^44^ Measures analyzed from the UK Biobank included total WMH volume, which was derived from both T1‐weighted and FLAIR images computed from the Brain Intensity Abnormality Classification Algorithm (BIANCA) of the FMRIB Software Library (FSL),^45^ and total intracranial volume (TIV) from the T1‐weighted scans using Freesurfer's automatic segmentation (aseg).^38^

### 2.4 Statistical analyses

Analyses were performed using the Statistical Package for the Social Sciences (SPSS) version 29^46^ and statistical figures were generated using R version 4.3.2.^47^ A *p* value (*p*) < 0.05 was used to indicate statistical significance. We observed a highly positive skew for WMH volume and a quantile–quantile plot that deviated from linearity. A Kolmogorov–Smirnov test confirmed a non‐normal distribution (D(15360) = 0.202, *p *< 0.001) and, in line with current practice,^45^ WMH volume was log‐transformed (natural log) for all analyses. Following transformation, 11 outliers were further identified using the median absolute deviation rule^48^ and were removed from all subsequent analyses.

Multiple linear regression tested the association between each dichotomized self‐reported sleep behavior and log‐transformed WMH volume. The first model (Model 1) examined this relationship while adjusting for TIV, age at imaging visit, sex, ethnicity (White vs non‐White), education (college or university degree vs no college or university degree), Townsend Deprivation Index, imaging center location, days between baseline and imaging visits, current depressed mood, and current alcohol use. The Townsend Deprivation Index is a single composite score of four sociodemographic variables (unemployment rate, non‐car ownership, non‐home ownership, and household overcrowding) standardized to have a mean of 0 and a SD of 1. Higher Townsend Deprivation Index scores indicate higher levels of socioeconomic deprivation, whereas lower scores indicate lower levels of deprivation. Current depressed mood was reported at the baseline visit to correspond to the participant's sleep behaviors. Participants were asked “Over the past two weeks, how often have you felt down, depressed or hopeless?” with available responses of “not at all,” “several days,” “more than half the days,” and “nearly every day” (f.2050). This was dichotomized in analyses to not at all versus several days to nearly every day. Current alcohol use was determined using proposed guidelines,^49^ where self‐reported alcohol consumption volume was multiplied by the alcohol content of the beverage (percentage) and divided by 0.6 ounces of alcohol per drink‐equivalent. We dichotomized these values of alcohol consumption as never versus moderate/high.

A follow‐up analysis (Model 2) additionally accounted for common vascular health and lifestyle factors associated with vascular brain aging and dementia risk to more specifically evaluate how each sleep behavior was related to WMH volume. This included self‐reported hypertension, diabetes, smoking status (never vs previous/current), weekly self‐reported moderate‐to‐vigorous physical activity, measured BMI, and apolipoprotein E (*APOE*) ε4 carrier status (non‐carrier vs carrier). Weekly moderate‐to‐vigorous physical activity was derived from their Metabolic Equivalent Task (MET) score on the International Physical Activity Questionnaire (IPAQ). Participant responses were dichotomized as not achieving versus achieving at least the recommended 150 minutes of walking or moderate activity per week or 75 minutes of vigorous activity per week (f.22036).^50^ Finally, we conducted a mutually adjusted model including all sleep behaviors that were independently associated with WMH volume in Model 2 to further examine their distinct contributions to vascular brain health. All *p* values were adjusted for multiple comparisons based on the false discovery rate (FDR) procedure^51^ and applied separately within each regression model (i.e., Model 1, Model 2, and the mutually adjusted model).

In addition, we conducted several sensitivity analyses to assess the robustness of our findings. First, when sleep duration was dichotomized only a relatively small number of individuals (*n* = 206; 0.9%) reported sleeping more than 9 h per day. To ensure these longer sleepers were not disproportionately influencing the results, we re‐analyzed Models 1 and 2 after excluding this subgroup for comparison. Second, although a majority of our sample was younger than 65 years of age (*n* = 12,963; 55.5%), there was still a substantial proportion of participants classified as older adults (≥65 years) according to NSF guidelines. As recommended sleep duration narrows to 7–8 h for this age group,^40^ we examined whether applying this restricted reference range would impact our findings. Sleep duration was recategorized as short (≤6 h; *n* = 4834), optimal (7–8 h; *n* = 17,208), and long (≥9 h; *n* = 1335). To facilitate comparison to the primary analyses, sleep duration was first dichotomized into optimal (7–8 h) versus suboptimal (less or more than 7–8 h) and was entered into Models 1 and 2. In addition, the larger number of long sleepers permitted pairwise linear regressions comparing short and long sleep separately to the 7‐8 h reference range across both models. For comparable FDR correction, each pairwise *p* value replaced the original sleep duration *p* value from the primary analyses and was tested alongside the other four sleep behaviors in the same five‐test FDR procedure. Third, we excluded individuals (*n* = 2293; 9.8%) who reported employment at least sometimes involving shiftwork at baseline (f.826) to reduce potential confounding due to disruptions in sleep behavior and related health outcomes associated with irregular work hours.^52^ Finally, we excluded individuals younger than 60 years of age at imaging (*n* = 8019; 34.3%) to examine the associations specifically in older adults. For each of these sensitivity analyses, *p* values were FDR‐corrected within the corresponding reduced sample.

## 3 RESULTS

**FIGURE 2:**
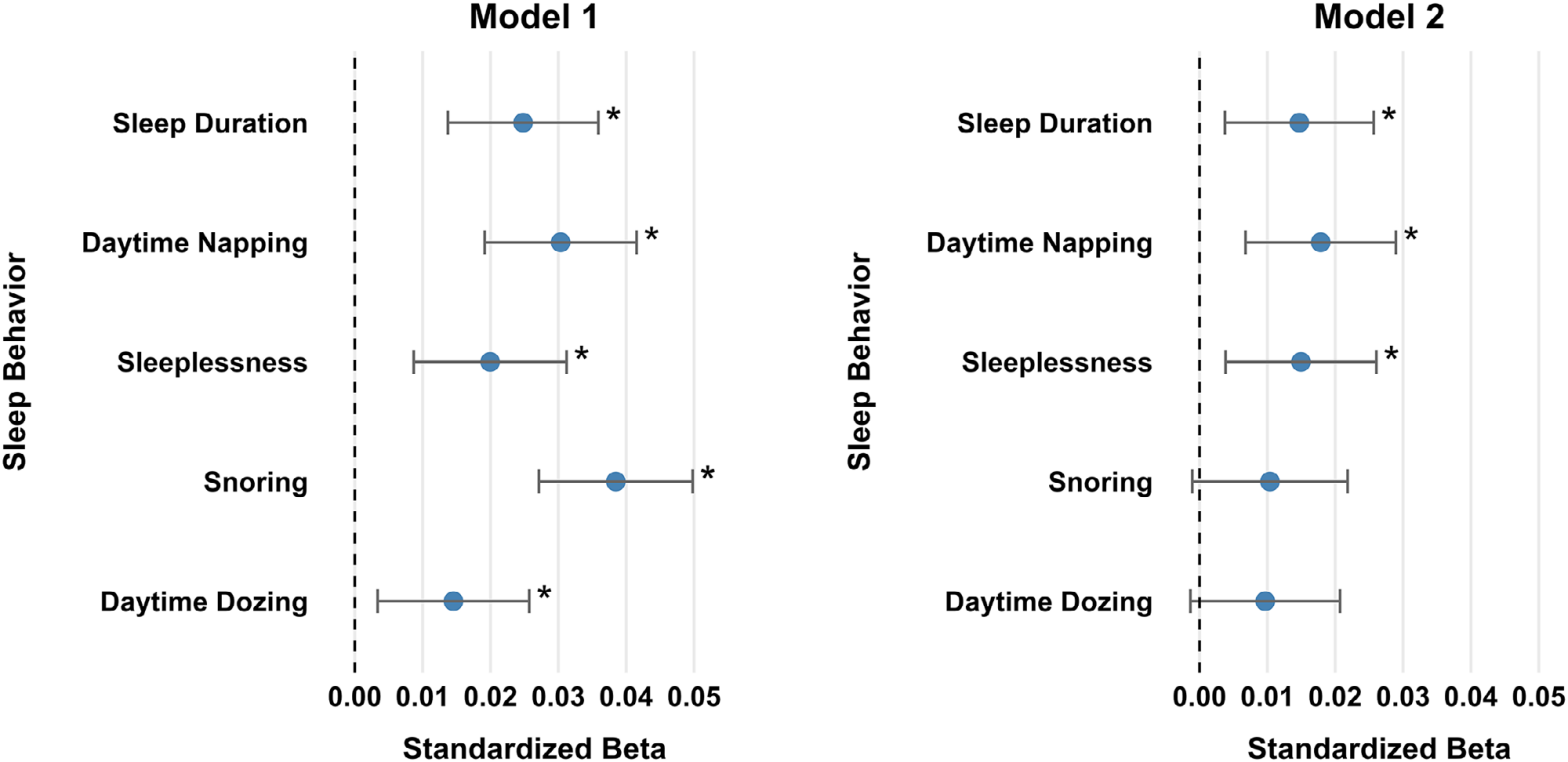
Forest plots comparing standardized beta coefficients for each sleep behavior across Model 1 and Model 2. NOTE: Standardized beta coefficients with 95% confidence intervals from multiple linear regression analyses between each self‐reported sleep behavior and log‐transformed white matter hyperintensity volume. Coefficients with confidence intervals not crossing zero are statistically significant at *p* < 0.05, and those remaining significant after false discovery rate correction using the Benjamini‐Hochberg procedure^51^ are indicated with an asterisk (*). Model 1 adjusted for total intracranial volume, days between baseline and imaging visits, imaging center location, age at imaging visit, sex, ethnicity, education, Townsend Deprivation Index, current depressed mood, and alcohol use at imaging. Model 2 additionally accounted for common vascular health and lifestyle factors associated with vascular brain aging and dementia risk, including self‐reported hypertension, diabetes, smoking status, weekly engagement in moderate‐to‐vigorous physical activity, measured body mass index, and apolipoprotein E (*APOE*) ε4 carrier status.

The present study included 23,377 individuals after implementing our inclusion and exclusion criteria (Figure 1). Participant characteristics of our final sample are provided in Table 1. When controlling solely for demographic, imaging acquisition, and clinical variables (Model 1), all sleep behaviors of interest were each associated with WMH volume assessed an average of 8.8 ± 1.7 (mean ± SD) years later, such that achieving less or greater than the 7‐9 h recommendation (*β* = 0.025, FDR*p* = 1.942E‐05), more frequent daytime napping (*β* = 0.030, FDR*p* = 2.749E‐07), increased sleeplessness (*β* = 0.020, FDR*p* = 6.498E‐04), the presence of snoring (*β* = 0.038, FDR*p* = 1.480E‐10), and more frequent daytime dozing (*β* = 0.015, FDR*p* = 0.011) corresponded to increased WMH volume (Table 2). When common vascular and lifestyle health factors were added to the model (Model 2), suboptimal sleep duration (i.e., below/above the 7‐9 h recommendation) (*β* = 0.015, FDR*p* = 0.014), more frequent daytime napping (*β* = 0.018, FDR*p* = 0.008), and greater sleeplessness (*β* = 0.015, FDR*p* = 0.014) remained associated with greater WMH volume (Table 2). Among these, engaging in more frequent daytime napping demonstrated the strongest association with WMH accumulation (Figure 2). However, the presence of snoring (*β* = 0.010, FDR*p* = 0.085) and increased daytime dozing (*β* = 0.010, FDR*p* = 0.085) were no longer significant. When the sleep behaviors that were independently significant in the fully adjusted model (sleep duration, daytime napping, and sleeplessness) were entered simultaneously into Model 2, all three remained associated with WMH volume (0.004 ≤ FDR*p'*s ≤ 0.025; Table 3), with daytime napping showing the relatively strongest effect.

**TABLE 1:** Participant characteristics (*n* = 23,377).

Variables	Mean (SD) or *n* (%)
*Demographic characteristics*	
Age, years	62.92 (7.44)
Sex, female	12,007 (51.4%)
Ethnicity, White	22,851 (97.7%)
Education, college or greater	11,847 (50.7%)
Townsend Deprivation Index	−2.00 (2.65)
*Imaging characteristics*	
Imaging center	
Cheadle	14,600 (62.5%)
Reading	2871 (12.3%)
Newcastle	5906 (25.3%)
Days between baseline and imaging visits	3211.74 (621.95)
WMH volume, mm^3^	4514.01 (5,657.12)
Total intracranial volume, mm^3^	1,550,744.20 (149,419.37)
*Clinical characteristics*	
Current depressed mood, several days to nearly every day	4656 (19.9%)
Alcohol use, moderate/high	19,963 (85.4%)
*Cerebrovascular risk factors*	
Smoking status, previous/current	8621 (36.9%)
Hypertension	4206 (18.0%)
Diabetes	529 (2.3%)
BMI	26.36 (4.29)
MVPA, at or above recommendation	18,974 (81.2%)
*APOE* ε4 carrier	6475 (27.7%)
*Sleep variables*	
Sleep duration, below/above the 7‐9 h recommendation	5040 (21.6%)
Daytime napping, sometimes/usually	8469 (36.2%)
Sleeplessness, sometimes/usually	16,642 (71.2%)
Snoring, yes	8374 (35.8%)
Daytime dozing, sometimes/often/all of the time	4535 (19.4%)

Abbreviations: *APOE*, apolipoprotein E; BMI, body mass index; MVPA, moderate‐to‐vigorous physical activity; WMH, white matter hyperintensity.

**TABLE 2:** Associations between sleep behaviors and white matter hyperintensity volume (*n* = 23,377).

Sleep behavior	*β*	B	95% CI	*p*	FDR*p*
***Model 1***					
Sleep duration	0.025	0.059	[0.033–0.085]	1.165E‐05	1.942E‐05
Daytime napping	0.030	0.062	[0.039–0.084]	1.100E‐07	2.749E‐07
Sleeplessness	0.020	0.043	[0.019–0.067]	5.198E‐04	6.498E‐04
Snoring	0.038	0.078	[0.055–0.101]	2.960E‐11	1.480E‐10
Daytime dozing	0.015	0.036	[0.008–0.063]	0.011	0.011
***Model 2***					
Sleep duration	0.015	0.035	[0.009–0.061]	0.009	0.014
Daytime napping	0.018	0.036	[0.014–0.059]	0.002	0.008
Sleeplessness	0.015	0.032	[0.008–0.056]	0.008	0.014
Snoring	0.010	0.021	[−0.002–0.044]	0.076	0.085
Daytime dozing	0.010	0.024	[−0.003–0.051]	0.085	0.085

*NOTE*: Multiple linear regression analyses between self‐reported sleep behaviors and log‐transformed white matter hyperintensity volume. Model 1 adjusted for total intracranial volume, imaging center location, days between baseline and imaging visits, age at imaging visit, sex, ethnicity, education, Townsend Deprivation Index, current depressed mood, and alcohol use at imaging. Model 2 additionally accounted for common vascular health and lifestyle factors associated with vascular brain aging and dementia risk, including self‐reported hypertension, diabetes, smoking status, weekly engagement in moderate‐to‐vigorous physical activity, measured body mass index, and *APOE* ε4 carrier status.

Abbreviations: *APOE*, apolipoprotein E; *β*, standardized regression coefficient; B, unstandardized regression coefficient; 95% CI, 95% confidence interval for B; FDR*p*, *p* value adjusted for multiple comparisons with false discovery rate following the Benjamini–Hochberg procedure.^51^

**TABLE 3:** Mutually adjusted model with significant sleep behaviors from Model 2 and white matter hyperintensity volume.

Sleep behavior	*β*	B	95% CI	*p*	FDR*p*
Sleep duration	0.014	0.034	[0.007–0.060]	0.012	0.018
Daytime napping	0.018	0.037	[0.014–0.059]	0.001	0.004
Sleeplessness	0.013	0.028	[0.003–0.052]	0.025	0.025

*NOTE*: Mutually adjusted multiple linear regression analysis including all self‐reported sleep behaviors that were independently significant, re‐entered simultaneously in Model 2, to predict log‐transformed white matter hyperintensity volume. Model 2 covariates included total intracranial volume, days between baseline and imaging visits, imaging center location, age at imaging visit, sex, ethnicity, education, Townsend Deprivation Index, current depressed mood, alcohol use at imaging, as well as common vascular health and lifestyle factors associated with vascular brain aging and dementia risk, including self‐reported hypertension, diabetes, smoking status, weekly engagement in moderate‐to‐vigorous physical activity, measured body mass index, and *APOE* ε4 carrier status.

Abbreviations: *APOE*, apolipoprotein E; *β*, standardized regression coefficient; B, unstandardized regression coefficient; 95% CI, 95% confidence interval for B; FDR*p*, *p* value adjusted for multiple comparisons with false discovery rate following the Benjamini–Hochberg procedure.^51^

We next examined the robustness of our findings through a series of sensitivity analyses. First, after excluding participants who reported sleeping more than the recommended 7–9 h (*n* = 23,171), the association between dichotomized sleep duration and WMH volume remained consistent in both Model 1 (*β* = 0.024, FDR*p* = 5.714E‐05) and Model 2 (*β* = 0.014, FDR*p* = 0.019). When sleep duration was recategorized according to NSF recommendations for older adults,^40^ suboptimal sleep (less or more than 7–8 h) remained associated with greater WMH volume in both Model 1 (*β* = 0.025, FDR*p* = 1.27E‐05) and Model 2 (*β* = 0.015, FDR*p* = 0.014). Separating suboptimal sleep into short (≤6 h) and long (≥9 h) durations revealed that short sleep was consistently associated with greater WMH volume (Model 1: *β* = 0.025, FDR*p* = 3.19E‐05; Model 2: *β = *0.015, FDR*p* = 0.018), whereas long sleep was not significant in either model (Model 1: *β* = 0.012, FDR*p* = 0.058; Model 2: *β* = 0.006, FDR*p* = 0.366). Third, after excluding participants with jobs that at least sometimes involved shiftwork (*n* = 21,084), all sleep behaviors remained associated with WMH volume in Model 1 and, consistent with results from the primary analysis, only obtaining less or greater than 7–9 h of sleep (*β *= 0.014, FDR*p* = 0.035), more frequent daytime napping (*β* = 0.019, FDR*p* = 0.008), and increased sleeplessness (*β  *= 0.017, FDR*p* = 0.013) remained associated in Model 2 (Table S1). Finally, when restricting the sample to individuals 60 years of age or older (*n* = 15,358; Table S2), all sleep behaviors of interest indicative of poorer sleep quality were associated with greater WMH volume in Model 1. After additionally adjusting for the vascular risk factors in Model 2, sleep duration outside the recommended range (*β* = 0.020, FDR*p* = 0.019), increased daytime napping (*β* = 0.022, FDR*p* = 0.019), and greater sleeplessness (*β* = 0.017, FDR*p* = 0.041) were associated with increased WMH volume (Table S3).

## 4 DISCUSSION

In a large cohort of healthy middle‐aged to older adults, we found that indicators of poor sleep quality, including failing to achieve the recommended 7‐9 h sleep duration (either below or above the NSF recommendation), increased daytime napping, greater sleeplessness, the presence of snoring, and more frequent daytime dozing, were prospectively associated with increased WMH volume after adjusting for demographic, imaging acquisition, and select clinical characteristics. Furthermore, the effects of sleep duration, daytime napping, and sleeplessness remained significant after further adjusting for common vascular health and lifestyle factors, suggesting that these behaviors may be distinctly associated with vascular brain aging, as reflected by increased WMH volume. In a mutually adjusted model containing all three of these sleep behaviors, each remained associated with WMH volume, suggesting a distinct contribution of each behavior, with daytime napping demonstrating the relatively strongest association. When individuals with jobs involving shiftwork were excluded, these associations persisted, indicating that these results were not driven by disruptions in sleep due to irregular working hours. In addition, when restricting the sample to individuals 60 years of age or older, the pattern of results remained unchanged, suggesting that the findings were not appreciably influenced by the younger middle‐aged participants. Together, these findings highlight that key aspects of sleep quality may reflect important characteristics associated with vascular brain health, distinct from other common risk factors known to influence brain aging and dementia risk.

Regular daytime napping was a relatively stronger predictor of WM lesion load at follow‐up after additionally accounting for common vascular health and lifestyle factors, as well as other sleep behaviors of sleep duration and sleeplessness. Few studies have specifically examined daytime napping as a distinct sleep behavior in relation to WM burden, rather than as part of an aggregate sleep quality metric. Prior work found that longer daytime sleep duration, relatively comparable to our daytime napping measure, was associated with greater WMH volume in a sample of 457 older adults.^33^ Our results corroborate and extend these findings, highlighting a key role of daytime napping in vascular brain aging and suggesting that WMH volume may serve as a useful marker for reflecting the impact of daytime napping on brain health and dementia risk. The relationship between daytime napping and cognitive impairment has been mixed, but evidence has begun to emerge suggesting that the effects of napping may be context dependent. More frequent and longer daytime naps (i.e., >60 min) have been associated with poorer cognitive performance^11^ and increased mortality risk,^13^ particularly due to AD.^12^ In contrast, shorter naps (i.e., ≤30–60 min) have been linked to a reduced risk of cognitive decline and dementia^8^, ^14^ and improved cognitive functioning.^53^ Although our findings add to understanding this complex relationship, further research into how these different aspects of napping relate to WMH volume is warranted to clarify the conditions under which napping may act as a potential risk or protective factor for dementia.

We found that suboptimal sleep duration, defined as obtaining less or more than the recommended 7–9 h per day according to NSF guidelines for adults,^40^ was associated with greater WMH volume. Although relatively few participants reported sleeping more than 9 h, sensitivity analyses indicated that this subset did not appreciably influence the results, which also remained consistent when applying the narrower 7‐8 h range recommended for older adults.^40^ Previous research has demonstrated a U‐shaped relationship between sleep duration and WMH lesion load, with both shorter (≤5–6 h/night) and longer (≥8–9 h/night) durations associated with greater WMH volume.^32^, ^34^, ^36^ Although our primary analyses could not fully evaluate this non‐linear pattern due to the small number of long sleepers, applying the 7‐8 h NSF recommendation for older adults showed that short sleep (≤6 h) was consistently associated with greater WMH volume, whereas long sleep (≥9 h) was not. Together, these findings suggest that the association between suboptimal sleep duration and increased WMH burden may be driven largely by short sleep, thereby underscoring the importance of maintaining sleep within recommended ranges. The robustness of our findings is strengthened by our use of a large, community‐dwelling cohort of middle‐aged to older adults, carefully applied exclusion criteria to reduce confounding from major medical, neurological, or psychiatric conditions, and adjustment for common vascular health and lifestyle factors, allowing a more precise examination of the distinct contribution of sleep duration to WMH volume.

Finally, we found that increased sleeplessness corresponded to greater WMH volume when accounting for all covariates. By excluding individuals with a reported diagnosis of insomnia, this measure reflected subclinical insomnia symptoms experienced by healthy adults. A previous study within the UK Biobank cohort did not find an association between sleeplessness and WMH volume,^54^ although there are several factors that may explain this discrepancy. First, participants were included solely based on having complete data, with no additional criteria applied. As we utilized more comprehensive inclusion and exclusion criteria to focus on healthy middle‐aged to older adults, our findings likely reflect the relationship between sleeplessness and WMH volume without the influence of major medical, neurological, and psychiatric comorbidities. In addition, in attempting to define the behavior as an indicator of insomnia, the prior study^54^ conducted a binary analysis that included those who “never” or “rarely” experienced sleeplessness versus those who “usually” experienced it, excluding individuals who reported “sometimes” experiencing sleeplessness, representing ≈48% of our cohort. Consequently, the results from the previous study primarily reflected an analysis of the extremes of sleeplessness, rather than the full continuum of this sleep behavior, potentially obscuring the understanding of its relationship with vascular brain health. Our findings support and emphasize the importance of further investigating this subclinical sleep behavior in relation to brain aging and dementia risk.

Another study similarly using the UK Biobank found that WMH volume served as a pathway through which an aggregate of sleep behaviors (e.g., snoring, daytime dozing, daytime napping) and morningness (reflecting a circadian rhythm chronotype) was associated with cognitive dysfunction.^55^ The authors proposed a cardiometabolic mechanism underlying this pathway, such that the sleep aggregate was associated with factors such as obesity, diabetes, dyslipidemia, and C‐reactive protein level, which in turn were associated with greater WMH volume and subsequent cognitive dysfunction. However, findings from our present study suggest that this relationship may be more complex, and that some sleep behaviors may be associated with WMH volume independent of such factors. The prior study included two measures in their composite (snoring and daytime dozing) that we found to become non‐significant after accounting for cardiovascular and lifestyle risk factors. Thus, their use of a latent sleep‐related symptom composite may not adequately capture the specific, complex relationship between each sleep behavior and WMH volume. Further investigation into potential mechanisms driving the association between distinct sleep behaviors and WM lesion load is warranted to clarify the variation in findings across studies and to examine potential causal pathways though which modifiable risk factors, such as sleep, relate to brain aging and dementia risk.

Our study has several strengths. First, we employed a large sample to evaluate the relationship between sleep behaviors and WMH burden. Distinct from most other studies, we applied extensive inclusion and exclusion criteria to minimize the influence of major medical, neurological, or psychiatric illness and sleep disorders. We also accounted for common vascular and lifestyle factors, allowing for a more specific evaluation of these sleep behaviors and vascular brain health within healthy middle‐aged to older adults. However, our study is not without limitations. First, although the study design was prospective, the observational nature of the design limits our ability to establish a causal relationship between sleep and WM lesion load. Longitudinal and intervention studies are needed to further assess directionality and to exclude the possibility of reverse causality. Second, many of our variables were derived from self‐reported data, thereby limiting the assessment of the sleep and cardiovascular variables, and introducing potential bias into the findings. Furthermore, the questions used to define the sleep behaviors were not part of a validated instrument, thus replication using additional validated and objective behavioral measures of sleep characteristics may be warranted. Third, although participants of the UK Biobank sample were randomly invited to participate in the imaging sub‐study, the invitees’ self‐selected participation could have introduced potential selection bias. Fourth, despite having a large sample and extensive covariates, we cannot exclude the possibility of residual or unmeasured confounding factors influencing the findings. Finally, the UK Biobank sample is racially and ethnically homogenous, potentially limiting the generalizability of these findings to more diverse populations.

In conclusion, after accounting for common vascular health and lifestyle factors, sleeping less or more than the recommended 7–9 h, engaging in more frequent daytime napping, and experiencing greater sleeplessness were each distinctly associated with greater WMH volume in a large cohort of community‐dwelling, healthy middle‐aged to older adults. These findings offer insight into a potential sleep‐related pathway that may have a unique association with vascular brain health and, in turn, dementia risk. Although future research is needed to clarify the underlying mechanisms and causality for these associations, our results underscore the importance of sleep quality and quantity that could help to bolster clinical recommendations and public health initiatives aimed at improving brain health outcomes in aging.

## CONFLICT OF INTEREST STATEMENT

The authors declare no conflicts of interest. Author disclosures are available in the supporting information.

## CONSENT STATEMENT

The study was approved by the National Health Service and the National Research Ethics Service, and all activities were performed in accordance with the Declaration of Helsinki. All participants provided written informed consent for UK Biobank participation.

## Supporting information




Supporting Information

Supporting Information
