# Supplementary material for: Associations of sleep behaviors with white matter hyperintensity volume in middle‐aged to older adults
Source: Alzheimers Dement. 2026 May 5;22(5):e71457. doi: 10.1002/alz.71457 (PMC13140584; doi:10.1002/alz.71457)
Supplement: Supplementary file 2 — Supporting Information [file ALZ-22-e71457-s001.docx]

**SUPPLEMENTARY MATERIALS** for

**Associations of sleep behaviors with white matter hyperintensity volume in middle-aged to older adults**

Madeline Ally, M.A., Daniel H. Aslan, Ph.D., M. Katherine Sayre, Ph.D., Pradyumna K. Bharadwaj, Ph.D., Silvio Maltagliati, Ph.D., Matthew D. Grilli, Ph.D., Mark H.C. Lai, Ph.D., Rand R. Wilcox, Ph.D., Yann C. Klimentidis, Ph.D., David A. Raichlen, Ph.D., Gene E. Alexander, Ph.D.

Supplementary Materials Included:

**Supplemental Table 1**. Associations between sleep behaviors and white matter hyperintensity volume excluding individuals with jobs at least sometimes involving shiftwork (n = 21,084).

**Supplemental Table 2**. Participant characteristics of those aged 60 years and older (n = 15,358).

**Supplemental Table 3**. Associations between sleep behaviors and white matter hyperintensity volume in those aged 60 years and older (n = 15,358).

**Supplemental Table 1.** Associations between sleep behaviors and white matter hyperintensity volume excluding individuals with jobs at least sometimes involving shiftwork (n = 21,084).

| Sleep Behavior | *β* | B | 95% CI | *p* | FDR*p* |  |
| --- | --- | --- | --- | --- | --- | --- |
| ***Model 1*** | | | | | | |
| Sleep duration | 0.023 | 0.056 | [0.028 – 0.084] | 7.693E-05 | 1.282E-04 |  |
| Daytime napping | 0.032 | 0.065 | [0.041 – 0.089] | 1.242E-07 | 3.104E-07 |  |
| Sleeplessness | 0.022 | 0.047 | [0.021 – 0.073] | 3.127E-04 | 3.908E-04 |  |
| Snoring | 0.036 | 0.074 | [0.049 – 0.098] | 2.736E-09 | 1.368E-08 |  |
| Daytime dozing | 0.013 | 0.032 | [0.003 – 0.061] | .031 | .031 |  |
| ***Model 2*** | | | | | | |
| Sleep duration | 0.014 | 0.033 | [0.005 – 0.060] | .021 | .035 |  |
| Daytime napping | 0.019 | 0.038 | [0.015 – 0.062] | .002 | .008 |  |
| Sleeplessness | 0.017 | 0.036 | [0.011 – 0.061] | .005 | .013 |  |
| Snoring | 0.009 | 0.018 | [-0.007 – 0.042] | .159 | .187 |  |
| Daytime dozing | 0.008 | 0.019 | [-0.009 – 0.048] | .187 | .187 |  |

**NOTE.** Multiple linear regression analyses between self-reported sleep behaviors and log-transformed white matter hyperintensity volume. Model 1 adjusted for total intracranial volume, days between baseline and imaging visits, imaging center location, age at imaging visit, sex, ethnicity, education, Townsend Deprivation Index, current depressed mood, and alcohol use at imaging. Model 2 additionally accounted for common vascular health and lifestyle factors associated with vascular brain aging and dementia risk, including self-reported hypertension, diabetes, smoking status, weekly engagement in moderate-to-vigorous physical activity, measured body mass index, and *APOE* ε4 carrier status.

Abbreviations: *β*, standardized regression coefficient; B, unstandardized regression coefficient; 95% CI, 95% confidence interval for B; FDR*p*, *P* value adjusted for multiple comparisons with false discovery rate following the Benjamini-Hochberg procedure.^51^

**Supplemental Table 2.** Participant characteristics of those aged 60 years and older (n = 15,358).

| Variables | Mean (SD) or n (%) |
| --- | --- |
| *Demographic Characteristics* |  |
| Age (years) | 67.32 (4.75) |
| Sex (female) | 7,550 (49.2%) |
| Ethnicity (White) | 15,099 (98.3%) |
| Education (college or greater) | 7,551 (49.2%) |
| Townsend deprivation index | -2.14 (2.57) |
| *Imaging Characteristics* |  |
| Imaging Center  Cheadle  Reading  Newcastle | 9,257 (60.3%)  2,049 (13.3%)  4,052 (26.4%) |
| Days between baseline and imaging visits | 3,250.62 (619.60) |
| WMH volume (mm^3^) | 5,620.02 (6,382.38) |
| Total intracranial volume (mm^3^) | 1,545,958.35 (150,791.43) |
| *Clinical Characteristics* |  |
| Current depressed mood (several days to nearly every day) | 2,676 (17.4%) |
| Alcohol use (moderate/high) | 13,067 (85.1%) |
| *Cerebrovascular Risk Factors* |  |
| Smoking status (previous/current) | 6,098 (39.7%) |
| Hypertension | 3,407 (22.2%) |
| Diabetes | 417 (2.7%) |
| BMI | 26.27 (4.10) |
| MVPA (at or above recommendation) | 12,578 (81.9%) |
| *APOE* ε4 carrier | 4,178 (27.2%) |
| *Sleep Variables* |  |
| Sleep duration (below/above the 7-9-hour recommendation) | 3,435 (22.4%) |
| Daytime napping (sometimes/usually) | 6,039 (39.3%) |
| Sleeplessness (sometimes/usually) | 11,361 (74.0%) |
| Snoring (yes) | 5,862 (38.2%) |
| Daytime dozing (sometimes/often/all of the time) | 3,388 (22.1%) |

Abbreviations: *APOE*, apolipoprotein E; BMI, body mass index; MVPA, moderate-to-vigorous physical activity; WMH, white matter hyperintensity.

**Supplemental Table 3.** Associations between sleep behaviors and white matter hyperintensity volume in those aged 60 years and older (n = 15,358).

| Sleep Behavior | *β* | B | 95% CI | *p* | FDR*p* |
| --- | --- | --- | --- | --- | --- |
| ***Model 1*** | | | | | |
| Sleep duration | 0.027 | 0.060 | [0.027 – 0.092] | 2.897E-04 | 4.828E-04 |
| Daytime napping | 0.036 | 0.068 | [0.041 – 0.096] | 1.322E-06 | 6.610E-06 |
| Sleeplessness | 0.021 | 0.043 | [0.012 – 0.074] | .006 | .008 |
| Snoring | 0.035 | 0.066 | [0.037 – 0.094] | 4.984E-06 | 1.246E-05 |
| Daytime dozing | 0.019 | 0.043 | [0.011 – 0.075] | .009 | .009 |
| ***Model 2*** | | | | | |
| Sleep duration | 0.020 | 0.043 | [0.012 – 0.075] | .007 | .019 |
| Daytime napping | 0.022 | 0.041 | [0.013 – 0.066] | .004 | .019 |
| Sleeplessness | 0.017 | 0.035 | [0.005 – 0.066] | .025 | .041 |
| Snoring | 0.009 | 0.018 | [-0.010 – 0.046] | .217 | .217 |
| Daytime dozing | 0.015 | 0.033 | [0.001 – 0.065] | .045 | .056 |

**NOTE.** Multiple linear regression analyses between self-reported sleep behaviors and log-transformed white matter hyperintensity volume. Model 1 adjusted for total intracranial volume, days between baseline and imaging visits, imaging center location, age at imaging visit, sex, ethnicity, education, Townsend Deprivation Index, current depressed mood, and alcohol use at imaging. Model 2 additionally accounted for common vascular health and lifestyle factors associated with vascular brain aging and dementia risk, including self-reported hypertension, diabetes, smoking status, weekly engagement in moderate-to-vigorous physical activity, measured body mass index, and *APOE* ε4 carrier status.

Abbreviations: *β*, standardized regression coefficient; B, unstandardized regression coefficient; 95% CI, 95% confidence interval for B. FDR*p*, *P* value adjusted for multiple comparisons with false discovery rate following the Benjamini-Hochberg procedure.^51^
